# Engineered Cyclodextrin-Starch Hydrogels for pH-Triggered Drug Release

**DOI:** 10.1021/acsomega.5c08972

**Published:** 2025-11-05

**Authors:** Adrielle C. Reis, Raphaela P. Guaringue, Vinicius M. Schaffka, Michele K. Lima-Tenório, Bárbara C. Fiorin, Adriano G. Viana, Ernandes T. Tenório-Neto

**Affiliations:** a Laboratory of Spectroscopy, Characterization and Modeling (LEsCaM), Department of Chemistry, State University of Ponta Grossa (UEPG), Av. General Carlos Cavalcanti, 4748, Ponta Grossa, Paraná, Brazil, CEP: 84030-900; b Laboratory of Multifunctional Polymeric Materials (LMPM), Department of Chemistry, State University of Ponta Grossa (UEPG), Av. General Carlos Cavalcanti, 4748, Ponta Grossa, Paraná, Brazil, CEP: 84030-900

## Abstract

Oral drug delivery systems must ensure that therapeutic agents reach the target site, minimizing premature release in the gastric environment. In this context, hydrogels have emerged as promising biomaterials due to their ability to swell, retain, and release drugs in response to environmental stimuli. However, optimizing hydrogel formulations, considering both the properties and the polymer–drug interactions, is challenging. Herein, starch and β-cyclodextrin (βCD) were modified with GMA to introduce polymerizable vinyl groups. The βCD was modified by transesterification, while the starch produced three distinct isomers due to additional epoxide ring-opening reactions. The modified molecules were cross-linked via free radical polymerization. A simplex-centroid design optimized the hydrogel formulations. The mathematical model could predict the composition that maximizes the difference between the swelling at pH 1.2 and 6.8. Scanning electron microscopy revealed that the porous size was dependent on the pH. The release studies of hydrocortisone indicated that only sample HG14 exhibited effective pH-responsive release, making it a promising candidate for intestinal drug delivery. Moreover, the release mechanism followed Fickian diffusion, ensuring controlled drug transport without burst release. These findings highlight the importance of optimizing the hydrogel formulation for achieving pH-responsive drug delivery and obtaining an advanced material for oral pharmaceutical applications.

## Graphical abstract

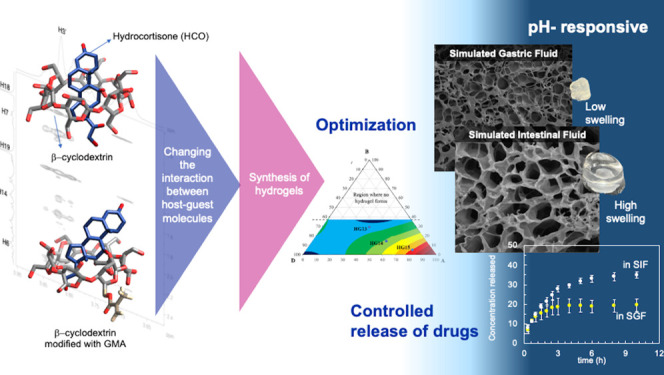

## 1 Introduction

Oral drug delivery systems represent the most widely used and convenient route for administering therapeutic compounds, owing to their noninvasive character, patient compliance, and reduced healthcare costs compared to parenteral administration. In particular, hydrogels have attracted attention as drug carriers due to their high-water absorption capacity, biocompatibility, and ability to respond to environmental stimuli, such as temperature, magnetic field, pH, and so on.^1–3^ Among the hydrogel systems, pH-sensitive matrices are particularly promising for oral drug delivery, as they can minimize premature drug release in the stomach, thereby promoting efficient drug absorption in the intestine.^1^

Molecules like cyclodextrins (CDs) appear suitable for enhancing the solubility of hydrophobic drugs. Accordingly, several works have exploited the potential of incorporating CDs in hydrogels.^5–7^ CDs are cyclic oligosaccharides known for their ability to form host–guest complexes with hydrophobic molecules.^8^ Among them, β-cyclodextrin (βCD) has been extensively investigated to improve the solubility and modulate the release of hydrophobic drugs. Despite these advances, designing βCD-based hydrogels that can simultaneously control swelling and drug release kinetics under pH variation remains a complex challenge.

Several strategies have been reported to integrate CDs into hydrogel matrices. For example, Clemence B.F. and coauthors developed a chitosan-based hydrogel with carboxymethyl-β-cyclodextrin, which improved the solubility and sustained release of Berberine hydrochloride.^9^ Recently, Jiang, S.Q. et al. grafted βCDs onto chitosan molecules to obtain a thermosensitive hydrogel for controlling the release of Quercetin.^6^ Finally, Lou, C., and colleagues prepared dialdehyde-β-cyclodextrin (DA-βCD) cross-linked with carboxymethyl chitosan (CMCS), demonstrating that the release of phenolphthalein could be modulated by adjusting the degree of cross-linking.^10^

Despite significant advances in hydrogel technology, there is still limited understanding of how the β-cyclodextrin (βCD) content and polymer composition influence pH-responsive behavior, particularly in systems designed for targeted intestinal drug delivery. This gap hinders the design of optimized formulations for controlled release of hydrophobic drugs in gastrointestinal environments.

Thus, this work aimed to synthesize and optimize pH-responsive hydrogels based on modified βCD and starch to evaluate their effects on the controlled release behavior of a model hydrophobic drug. Hydrocortisone (HCO) was selected as the model drug due to its low aqueous solubility and therapeutical importance. HCO is a corticosteroid widely used for treating inflammatory disorders. Its long-term use may lead to several side effects, including Cushingoid appearance, hirsutism, and impotence.^4^ Thus, the controlled release of HCO is essential to maximize therapeutic efficacy while reducing side effects. According to our recent studies, the starch-based hydrogel is nontoxic and safe for use as a biomaterial.^11–13^ To enhance HCO solubility, the βCD was incorporated into the hydrogels. As a strategy, βCD was chemically modified with glycidyl methacrylate (GMA), allowing covalent incorporation into the hydrogel network, rather than acting as a dispersed additive. We hypothesize that incorporating βCD into starch-based hydrogels may influence their swelling properties, thereby altering the drug release kinetics. Thus, optimizing the hydrogel composition is necessary for effective hydrophobic drug delivery.

The hydrogels were synthesized by combining the modified polysaccharides with *N, N*-dimethylacrylamide and sodium acrylate. The latter component modulates pH responsiveness, water absorption, and drug diffusion.^14^

We investigated how modified βCD affects both inclusion-complex formation and the structural and functional properties of starch-based hydrogels. A simplex-centroid design was used to optimize the formulations that minimize swelling in acidic conditions and maximal response in neutral pH. Additionally, other hydrogel properties (including swelling behavior, drug release mechanism, and their relationship with pH and polymer composition) were studied. The results demonstrated the potential applications of these hydrogels for oral drug delivery.

## 2 Experimental Section

### 2.1 Materials

Starch from potato (*M*_w_ = 10.57 ± 1.5 kDa; branching degree 14.78 ± 0.25%), β-cyclodextrin ≥ 95%, glycidyl methacrylate (GMA) 97%, acrylic acid 99%, potassium persulfate ≥ 98%, hydrocortisone (HCO) ≥ 98%, N, N-dimethyl acrylamide (DMAAm) 99% were purchased from Sigma-Aldrich. Dimethyl sulfoxide (DMSO) P.A. and sodium hydroxide P.A. were acquired from Dinâmica. Sodium chloride and hydrochloric acid were acquired from Reatec. Potassium phosphate monobasic was obtained from Perquim. Acetone P.A., ethanol 99.5% were purchased from Neon. Deuterium oxide (D_2_O), D, 99.9%, dimethyl sulfoxide-D_6_, D, 99.9%, 3-(trimethylsilyl) propionic-2,2,3,3d4 acid sodium salt (TMS-p D4) were acquired from Cambridge Isotope Laboratories, Inc. All reactants were used without further purification.

### 2.2 Preparation of Buffer Solutions

The effects of gastrointestinal fluids on the release of hydrocortisone were investigated by *in vitro* experiments, using simulated fluids without enzymes. They were prepared following the general procedure described in our previous work.^4^ The simulated gastric fluid (SGF, pH 1.2) comprised 2.0 g L^–1^ of sodium chloride and 0.08 M of hydrochloric acid. The simulated intestinal fluid (SIF, pH 6.8) comprised 0.62 g L^–1^ of sodium hydroxide and 6.8 g L^–1^ of potassium phosphate monobasic.

### 2.3 Chemical Modification of Polysaccharides with GMA

#### 2.3.1 Modification of Starch

The starch modification was carried out following the methodology of Lima-Tenório, M. et al.^11^ Five grams of starch were dissolved in 100 mL of DMSO at 90 °C, under continuous stirring. Then, the temperature was reduced to 60 °C, and NaOH was added to adjust the pH to c.a. 10.5. Subsequently, two milliliters of GMA were added into the reactor, and the reaction proceeded for 24 h. Afterward, the resulting product was precipitated, washed with ethanol, and freeze-dried for 12 h.

#### 2.3.2 Modification of βCD

In a three-necked flask containing 50 mL of 0.2 M NaOH, ten grams of βCD (8.81 × 10^–3^ mol) was added. The mixture was heated up to 60 °C, and stirred until its homogenization. Subsequently, 2.5 g (17.6 × 10^–3^ mol) of GMA was added into the reactor, keeping it under stirring at 60 °C in an oil bath for 5 h. After the reaction time, the mixture was cooled to room temperature and stirred overnight. The resulting whitish suspension was centrifugated, washed extensively with acetone, and dried in a vacuum oven at 25 °C. The final product was characterized by ^1^H NMR.^15^

### 2.4 Synthesis of Hydrogels (HG)

Before gelation, the neutralization of acrylic acid with a strong base (NaOH) was performed to obtain the respective acrylate salt. The use of acrylate salt is preferable to avoid overheating. Briefly, an equimolar amount of acrylic acid and NaOH were solubilized in acetone under continuous stirring. After 6 h, the whitish suspension (acrylate salt) was vacuum-filtered, and dried in a ventilated oven at 35 °C for 48h.

The hydrogels were obtained in 1.5 mL Eppendorf tube through a mixing 500 μL of modified starch (0.1 g mL^–1^), and desired amount of DMMAm (density of 0.962 g mL^–1^), modified β-cyclodextrin (0.010 g mL^–1^), acrylate salt (0.3 g mL^–1^) (see Table 1). The mixture was heated up to 70 °C using a heat block (FINEPCR model ALB64). After, 6.0 μL of potassium persulfate (0.5 g mL^–1^) was added into the tube followed by vigorous mixing. The total volume of each hydrogel was 1000 μL. The polymerization took place for 30 min at 70 °C, and then, the sample was left to cool to room temperature for another 30 min. The hydrogels were dried in a ventilated oven, at 40 °C, and stored for further use.

**Table 1:** Compositions for the Simplex-Centroid Design

	volumes (μL)			composition in mass (%)		
sample	D	B^a^	A^a^	D	B^a^	A^a^
HG1	5.0	481	8.0	40	40	20
HG2	2.0	476	16	17	41	42
HG3	157	0	337	60	0	40
HG4	85	0	409	40	0	60
HG5	14	464	15	60	20	20
HG6	9.0	454	30	39	20	41
HG7	5.0	445	44	21	20	59
HG8	274	0	220	80	0	20
HG9	20	453	20	65	15	20
HG10	12	438	44	40	15	45
HG11	39	402	54	65	7	28
HG12	27	383	84	47	7	46
HG13	20	433	41	54	12	34
HG14	34	373	87	52	6	42

a (B) modified β-cyclodextrin (0.01 g mL^–1^), acrylate salt (0.3 g mL^–1^).

#### 2.4.1 Experimental Design

The investigated formulations were designed to exhibit minimal swelling in an acidic medium and significant swelling at a neutral pH. To identify and optimize the best component proportions, a simplex-centroid mixture design was employed. The variables considered in the design were the modified β-cyclodextrin (B), DMMAm (D), and acrylate salt (A), while the amounts of modified starch and sodium persulfate were kept constant across all formulations. The investigated experimental range was selected to ensure that the resulting hydrogels remain biocompatible.^11,13^

The total volume of reactants used in each formulation was 1000 μL. The amounts of modified starch and potassium persulfate were fixed at 500 and 6 μL, respectively. Only the volumes of D, B, and A were varied, and their percentage compositions by mass were calculated based solely on these three components. This approach was adopted to due to the low solubility of modified βCD, which required precise volume adjustments while preserving the desired mass ratios.

The response variable was defined as ΔSwelling, which corresponds to the difference between the maximum swelling in SIF and SGF. The designed compositions are presented in Table 1.

Statistical analysis of variance (ANOVA), contour graphs, and the empirical model were performed using Excel software. Model accuracy was validated by assessing the correlation between the experimental and predicted results. The significance of the factors and their interactions was determined using an F-test at a 95% confidence level. To minimize systematic errors, the experimental runs were conducted in a randomized order.

### 2.5 Characterizations

#### 2.5.1 Nuclear Magnetic Resonance (NMR)

All ^1^H NMR spectra were acquired at 298 K on a Bruker AVANCE III spectrometer operating at 9.4 T, observing 1H at 400.13 MHz and 13C at 100.13 MHz, and equipped with a broadband probe. For both the inclusion analysis of HCO into βCD and the stoichiometry determination, spectra were acquired in D_2_O (TSP-*d*_4_ 5 mM) with 64k data points over a spectral width of 20 ppm, using a single 30° excitation pulse, a 1 s relaxation delay, and 128 scans. The stoichiometry of the βCD/HCO complex was determined by the continuous variations’ method. A stock solution of βCD and HCO was prepared and mixed to a constant concentration of 2 mM, varying proportion of host (βCD) and guest (HCO) to complete the molar ratio (r) range *r* = [β*CD*]/([β*CD*] + [*HCO*]). The data were plotted in the form of r versus Δδ­[βCD] (Job’s Plot).^16,17^ The spectra were referenced to the HDO signal at 4.70 ppm.

The 2D ROESY spectra were acquired in phase-sensitive mode with continuous-wave solvent suppression (roesyphpr.2) to determine the spatial arrangement of the βCD/HCO complex. Measurements were performed using an equimolar (1:1) sample with a spectral width of 10 ppm, 2k data points in the F2 dimension, and 512 data points in the F1 dimension. The acquisition parameters included a relaxation delay of 2 s, a mixing time of 200 ms, and 32 scans.

The modification of starch and βCD with glycidyl methacrylate (GMA) was characterized by ^1^H NMR spectroscopy using the same pulse sequence and acquisition parameters described above. However, for the starch/GMA sample, spectra were acquired in DMSO-*d*_6_ (TMS 0.05%) with 128k data points to enhance the signal-to-noise ratio. The determination of the modification degree of GMA/βCD also had an exception; the relaxation delay was set to 60s for the relative quantification of the substitution degree.

##### 2.5.1.1 Branching Degree (BD) and Molecular Weight of Starch

The BD was calculated following the procedure described by Boccia et al.^18^ The data ^1^H NMR spectroscopy using DMSO-*d*_6_ as solvent. The BD of starch was calculated from the relationship between the integral values of the anomeric proton signals α – 1,4 and α – 1,6:

$$BD\left(\%\right)=\frac{I_{\alpha -1,6}}{I_{\alpha -1,6}+I_{\alpha -1,4}}\times 100$$ 1

where *I*_α – 1,6_ and *I*_α – 1,4_ are respectively the ^1^H integral values of proton signals from internal α-1,6 in terminal nonreducing ends (branched), and from linear α-1,4 linkages.

The average molecular weight of starch was estimated by Diffusion-ordered NMR spectroscopy (DOSY) using the method described by Viel et al. and Tooley et al.^19,20^ Acquisition and processing parameters, and theoretical description of the methodology are reported in the Supporting Information.

#### 2.5.2 Scanning Electron Microscopy

The hydrogels were swollen into a buffer solution (pH 6.8 or pH 1.2) until reaching the equilibrium. Then, the swollen HGs were withdrawn from the solution, immediately frozen in liquid nitrogen, and lyophilized for 48 h. It is supposed that under these conditions, the internal structure of the hydrogels remains unchanged. Then, the lyophilized materials were fractured to expose their internal structure. Afterward, the fractured HGs were placed into stubs, and their surfaces were coated with a thin layer of gold. The SEM images were obtained on a Scanning Electron Microscope Tescan (Vega 3) by applying an acceleration voltage of 15 kV and a current intensity of 30 μA.

#### 2.5.3 Swelling Measurements

Swelling kinetics of samples were investigated by immersing a known amount of dried hydrogel into a simulated fluid (SGF or SIF) at 37 °C. Then, at specific time intervals, the hydrogels were withdrawn from the buffer solution, their surface was wiped off carefully to remove the excess water droplets, and the samples were weighed. After, the HGs were returned to the buffer solution. The data of swelling kinetics is shown as an average of three different experiments (*n* = 3). The swelling degree (S) was calculated from eq 2:

$$S=\frac{w_{t}-w_{o}}{w_{o}}$$ 2

were *w*_*t*_ and *w*_*o*_ is the water content within the hydrogel at any time, and the initial mass of dried hydrogel, respectively.

#### 2.5.4 *In Vitro* Drug Release

The experimental assays were performed using hydrogels HG13, HG14, and HG15. In order to ensure a uniform comparison and isolate the influence of βCD during the release process, we adopted an *in situ* loading approach, in which a fixed amount of 1.0 mg of HCO was incorporated in each sample during hydrogel synthesis (composition described in Table 1). After synthesis, the hydrogels were dried in a ventilated oven at 40 °C for 24 h. The resulting dried hydrogels had weights ranging from 86 to 170 mg.

The dried and HCO-loaded hydrogels were immersed in a glass reactor containing 25 mL of simulated fluid (pH 1.2 or pH 6.8) to mimic the physiological conditions of the gastrointestinal tract. All experiments were performed in triplicate. The experiments were conducted at 37 °C, under controlled agitation and uniform homogenization. To ensure the sink conditions, the release system was designed to release a maximum of 40 μg mL^–1^ of HCO, while the saturation concentration of HCO ranges from 200 to 300 μg mL^–1^.

At predefined time intervals, 1.0 mL aliquots of the dissolution medium were collected and analyzed by UV–vis spectrophotometry at 247 nm, corresponding to the maximum absorption wavelength of hydrocortisone.^4,21^ Then, the aliquots were returned to the medium to maintain a constant volume throughout the experiment.

The fractional drug release was quantified through the relationship between the temporal variation in HCO concentration with the total amount initially incorporated into the hydrogel. The release mechanism was determined by the Korsmeyer-Peppas equation, which correlates the fractional release (*C*_*t*_/*C*_∞_) as a function of time:

$$\frac{C_{t}}{C_{\infty }}=kt^{n}$$ 3

where *k* is a constant incorporating structural and geometric characteristics of the sample, and *n* is a diffusional exponent related to the release mechanism.^22,23^

## 3 Results and Discussion

### 3.1 Characterization of Modified Starch and Modified βCD with GMA

The chemical modification of starch was confirmed by the presence of GMA protons in the modified starch spectrum (Figure 1). The signals at 6.07 and 5.69 ppm were associated with the vinylic protons of GMA H1 and H1’, respectively.^11^ The signal corresponding to the methylene group (H2) was observed at 1.89 ppm. Furthermore, the absence of signals at 2.8 and 2.6 ppm, which correspond to the epoxide ring of GMA, confirms that the modification occurred via the epoxide ring-opening mechanism.^24^

**Figure 1:**
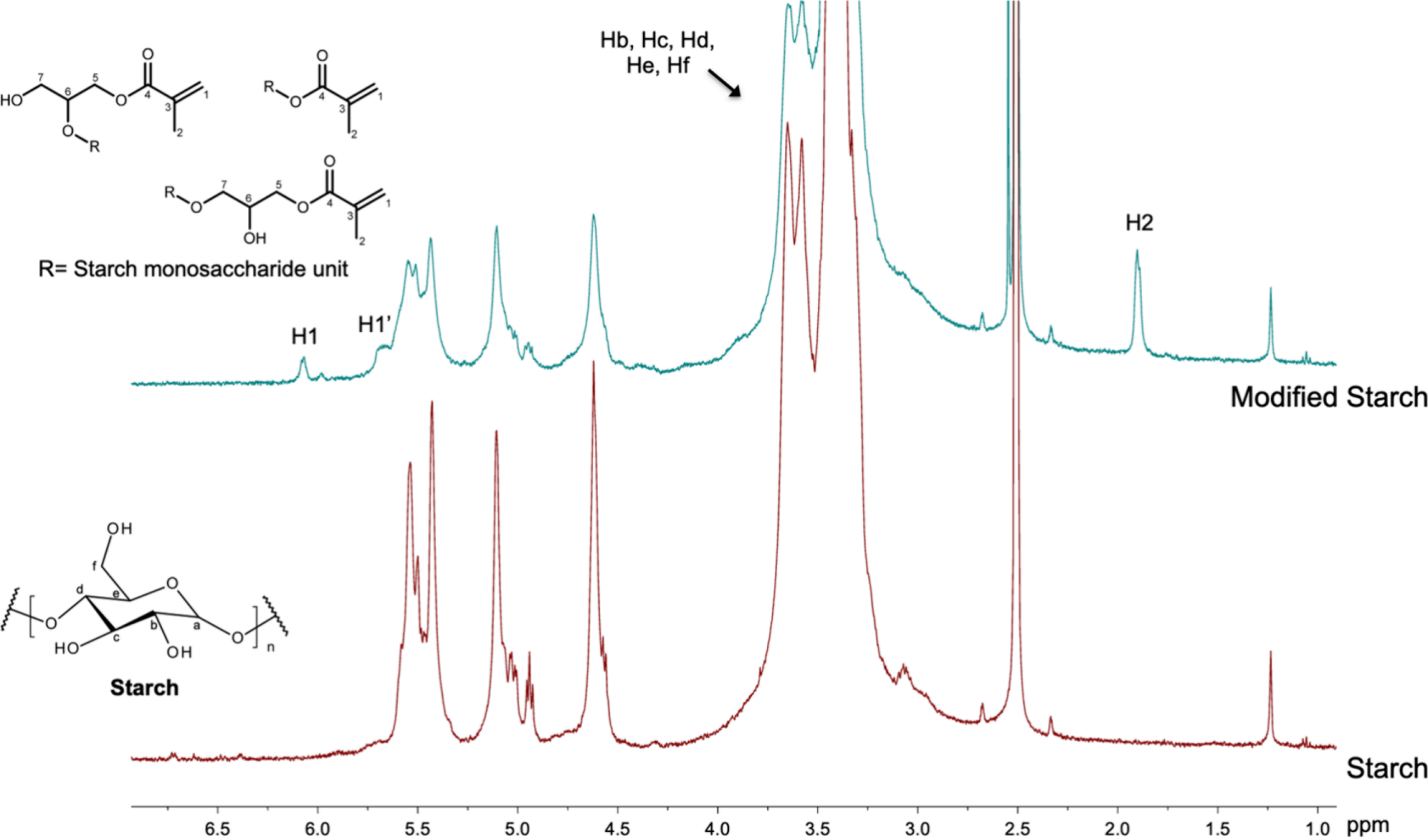
^1^H NMR spectra of starch and the modified starch with GMA.

Under the reaction conditions employed, no selectivity toward a specific product was observed, as the modification can lead to the formation of three distinct products: (i) 3-methacryloyl-1-glyceryl ether of starch, (ii) 3-methacryloyl-1-glyceryl ether of starch, and (iii) methacrylate of starch.^25^ In addition, the short T2 relaxation time of starch macromolecules increases spectral line broadening, which harms signal assignment of all products formed.

Figure 2 shows the spectrum of the modified βCD. The signals corresponding to the vinylic protons (Ha and Hb) at 6.09 and 5.97 ppm and the methyl group at 1.85 ppm were associated with GMA. Furthermore, the results of the ^1^H–^13^C HMBC spectrum suggest correlations between the signals of the vinylic protons Ha and Hb to the carbonyl group, vinylic carbon, and methyl carbon at 170.55, 137.10, and19.10 ppm, respectively (see Supporting Information, Figure S1). Moreover, the integrated area of ^1^H signals was 1:7, indicating that only one of the seven monosaccharide units of βCD was modified by GMA. As illustrated in Figure 2, such results indicate that only a single modification product is formed, occurring in the −OH groups from C6’. All ^1^H and ^13^C NMR assignments, as well as the ^1^H–^13^C HMBC correlations, are presented in the Supporting Information.

**Figure 2:**
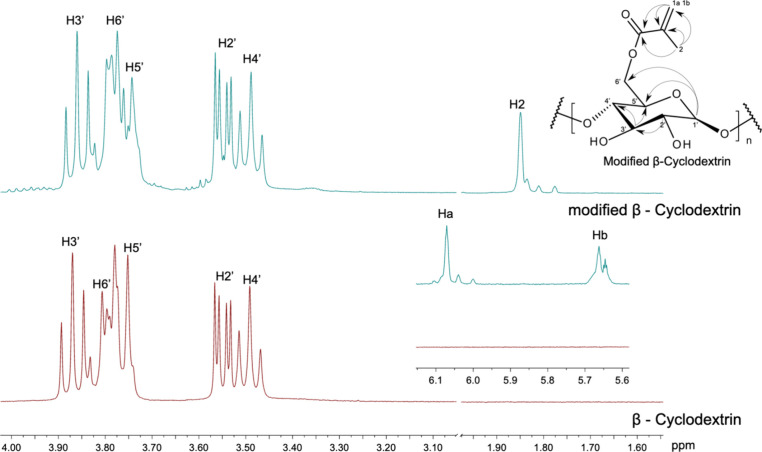
^1^H NMR spectra of βCD and modified βCD with the ^1^H–^13^C HMBC. The correlations are presented by arrows.

### 3.2 Inclusion Studies

Hydrocortisone (HCO) was chosen as a drug model due to its poor solubility. The βCD is widely used as a molecular host, improving the solubility and bioavailability of guest molecules. To assess the inclusion of HCO into the βCD cavity, the ^1^H NMR measurements were employed. The spectra of the pure compounds were recorded (Figure 3A), followed by their mixtures across the full range of molar ratios (0 < *r* < 1) to determine the stoichiometry of the inclusion complex (Figure 3B). Evidence of the inclusion was based on chemical shifts in the signals of both βCD (host) and HCO (guest) protons.

**Figure 3:**
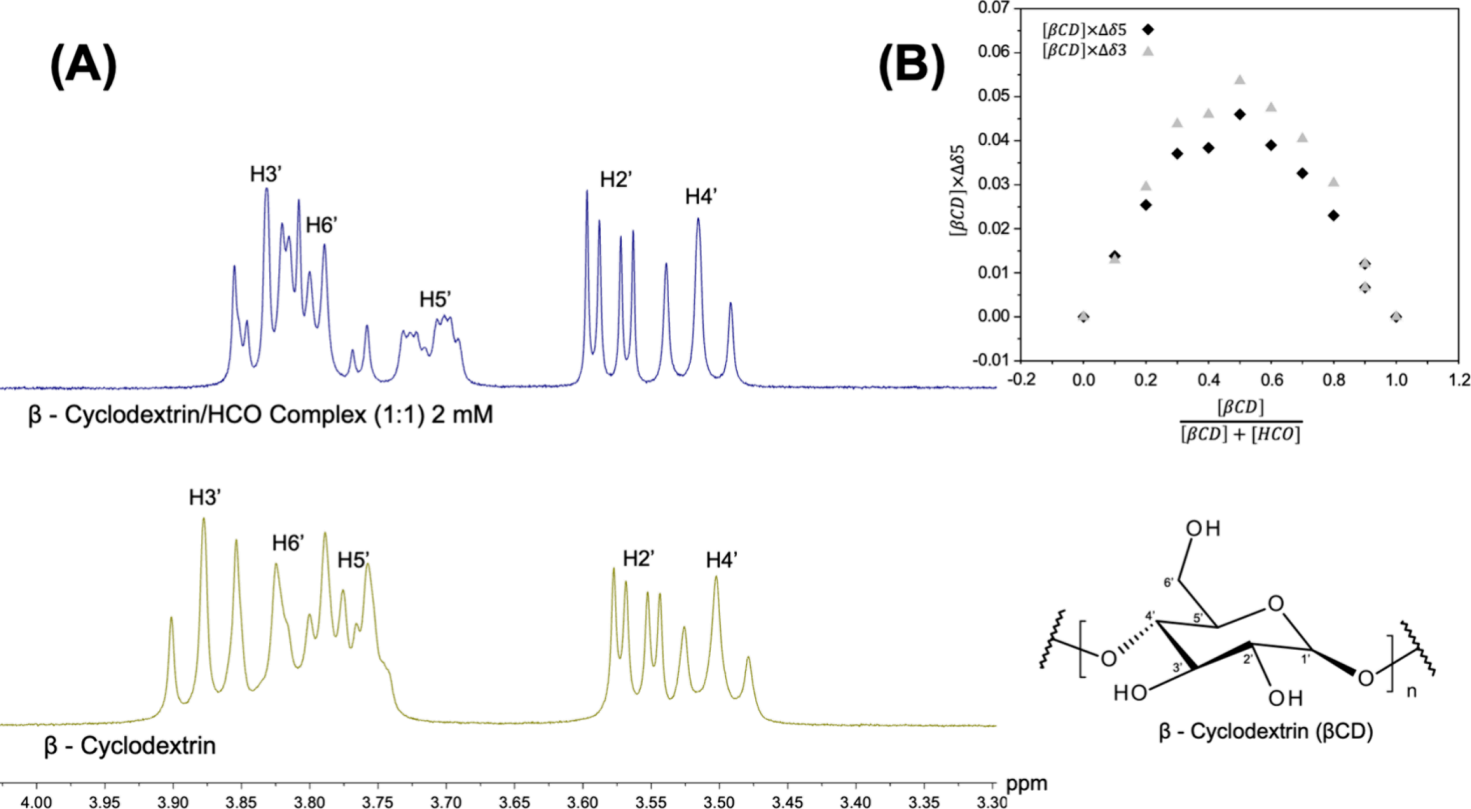
(A)^1^H NMR spectra of βCD/HCO inclusion complex and βCD (A). The Job’s plot of the βCD/HCO complex (B).

The upfield shifts are observed in the H3′ and H5′ protons of βCD, located inside its cavity. These chemical shifts, associated with the absence of new signals, provide evidence for forming the βCD/HCO complex. The protons outside the cavity (H1’, H2’, and H4’) show minimal shift changes. Additionally, downfield shifts in all HCO protons can be observed mainly due to complexation.^17,26,27^

The stoichiometry of the βCD/HCO complex was determined by the continuous variation method (Figure 3B). The Job’s Plot was constructed for both βCD and HCO protons. They show a maximum at r = 0.5, indicating the formation of a 1:1 complex.^27^ We found that independently of the chemical modification, the stoichiometry of complex formation is not altered.

#### 3.2.1 ROESY of HCO/βCD Complex

Figure 4 shows the 2D ROESY spectra of HCO and βCD in a proportion 1:1. This approach provides valuable information about the spatial proximity of atoms by the cross-peaks, due to the nuclear Overhauser effect (NOE). The interactions between the protons of HCO and βCD molecules were observed, indicating the inclusion of the drug within the βCD cavity. The methylene groups H18 and H19 strongly interact with the H3′ and H5′, suggesting that hydrocortisone is fully included in the cavity. This finding is contrary to previous studies which have indicated that regarding hydrocortisone, only partial inclusion complexes could be formed, in which they only detected ROE signal to the H3′ proton of β-CD.^27,28^ Moreover, aliphatic protons of HCO, such as H6, H7, and H14, showed cross-peaks with H3′. At the same time, no correlations with H5′ were found. This result suggests that HCO is faced and closer with methyl groups inside the βCD cavity, as the strength of ROE signals is proportional to the inverse sixth power of the distance between atoms.^29^ Based on these results, we proposed a schema suggesting the complex structure (Figure 4B).

**Figure 4:**
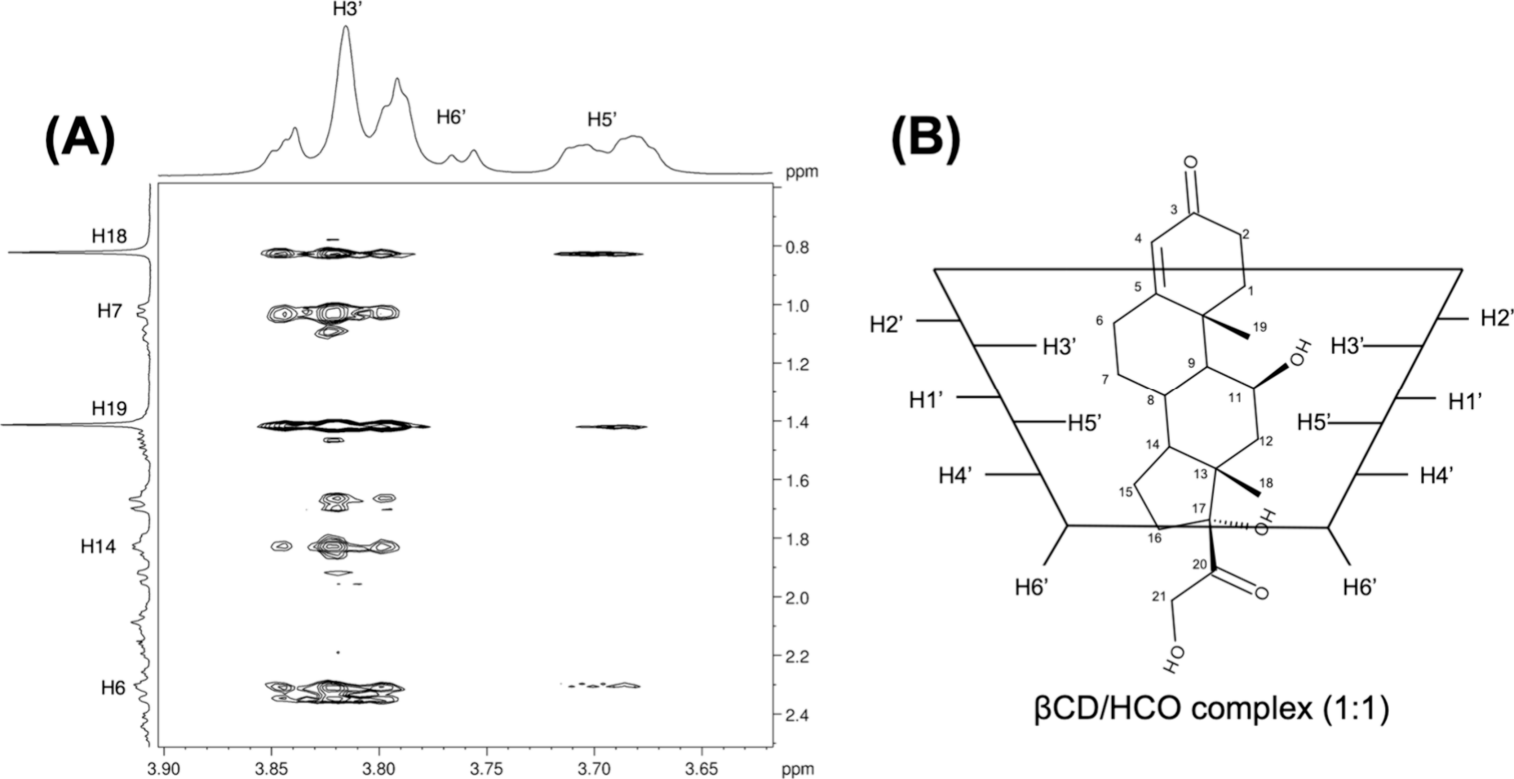
2D ROESY spectrum of Hydrocortisone/βCD complex (A) and a schema suggesting the structure of the complex (B).

The presence of GMA on the modified βCD, changes the interaction with HCO. The 2D ROESY spectra of modified βCD/HCO is shown in Figure 5A.

**Figure 5:**
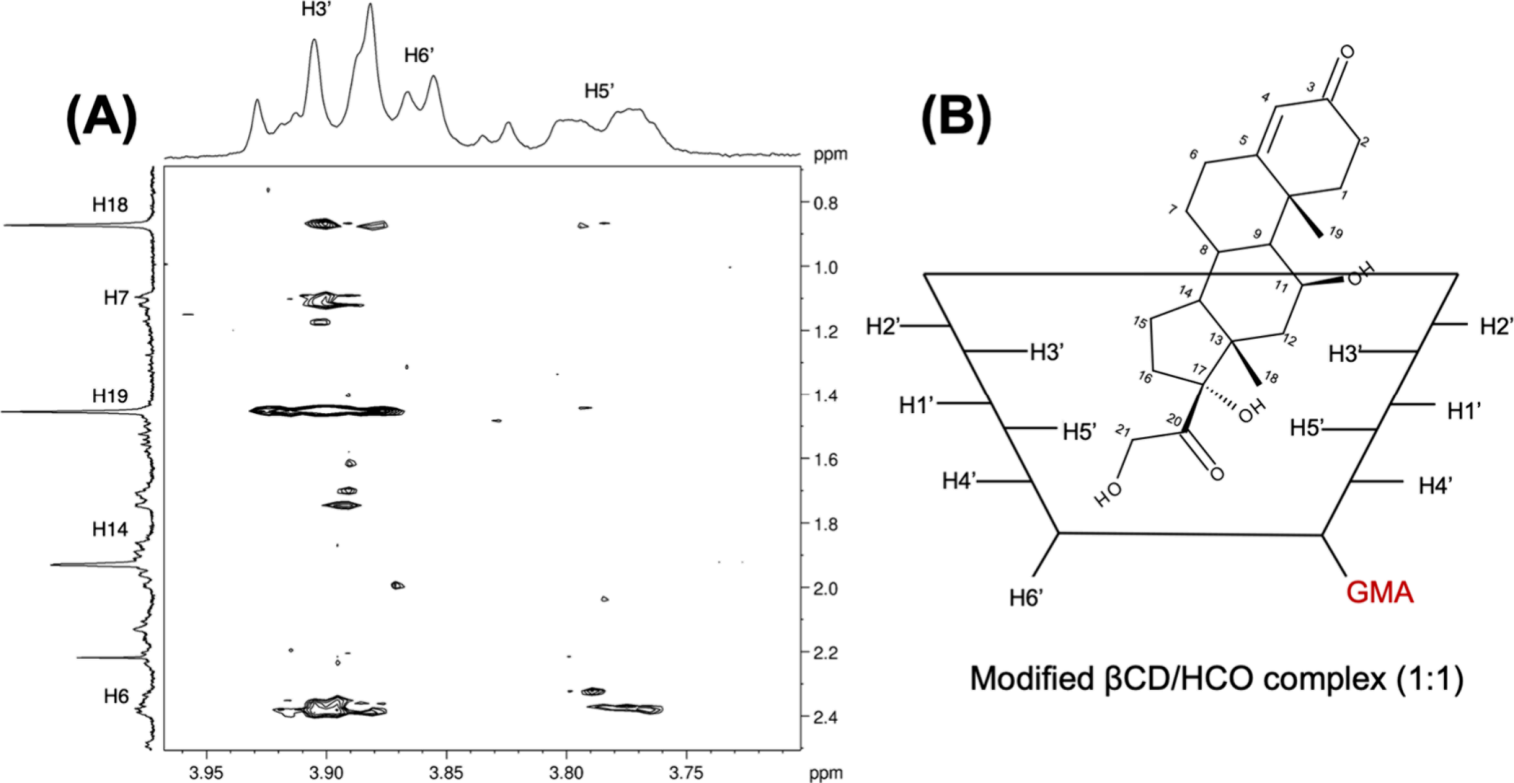
2D ROESY spectrum of Hydrocortisone/modified βCD complex (A) and the proposed structure of the complex (B).

In this Figure, there are cross-peaks between methylene groups H18, and H19 of HCO, with H3′, but with a lower intensity. Fereidounpour, P., and colleagues evaluated the effect of cyclodextrin substitution in the binding free energy of cyclodextrin-steroid complexes.^30^ The results showed that increasing the degree of βCD substitution or modifying the cavity size with larger groups reduced complex stability. These modifications made the complexes less stable, than those formed with native βCD. Thus, due to the presence of GMA, the complex structure is slightly changed. A schema is presented in Figure 5B.

The association constant (Ka) is an index that indicates how easily the complex can be formed. Usually, the higher the Ka value, the better the interaction. Thus, a simulation of a supramolecular titration based on the chemical shift changes in H3′ and H5′ was performed.^31^ The Ka results for βCD and modified βCD were 3903.47 M^–1^ and 1424.31 M^–1^, respectively. These results agree with the ROESY observations, indicating that the insertion of GMA in the βCD structure, changes the dynamics of the βCD/HCO complex formation by decreasing the affinity of HCO for the βCD cavity. It is important to highlight that, even with a decrease in affinity, the complex stoichiometry between modified βCD and HCO is the same.

### 3.3 Optimization of the Hydrogel Composition by a Simplex-Centroid Design

In oral drug delivery systems, where the focus is to release the drug in an intestine medium, the hydrogel must have a lower swelling ratio in an acidic environment (protecting the drug). On the other hand, it must show the highest swelling degree in the intestinal fluid to release the drug, promoting its absorption.^32,33^

For such a purpose, we investigated and optimized the hydrogel composition, evaluating the variation in swelling (ΔSwelling) under different pH values (1.2 and 6.8). Initially, the blue region located in Figure 6A was first investigated.

**Figure 6:**
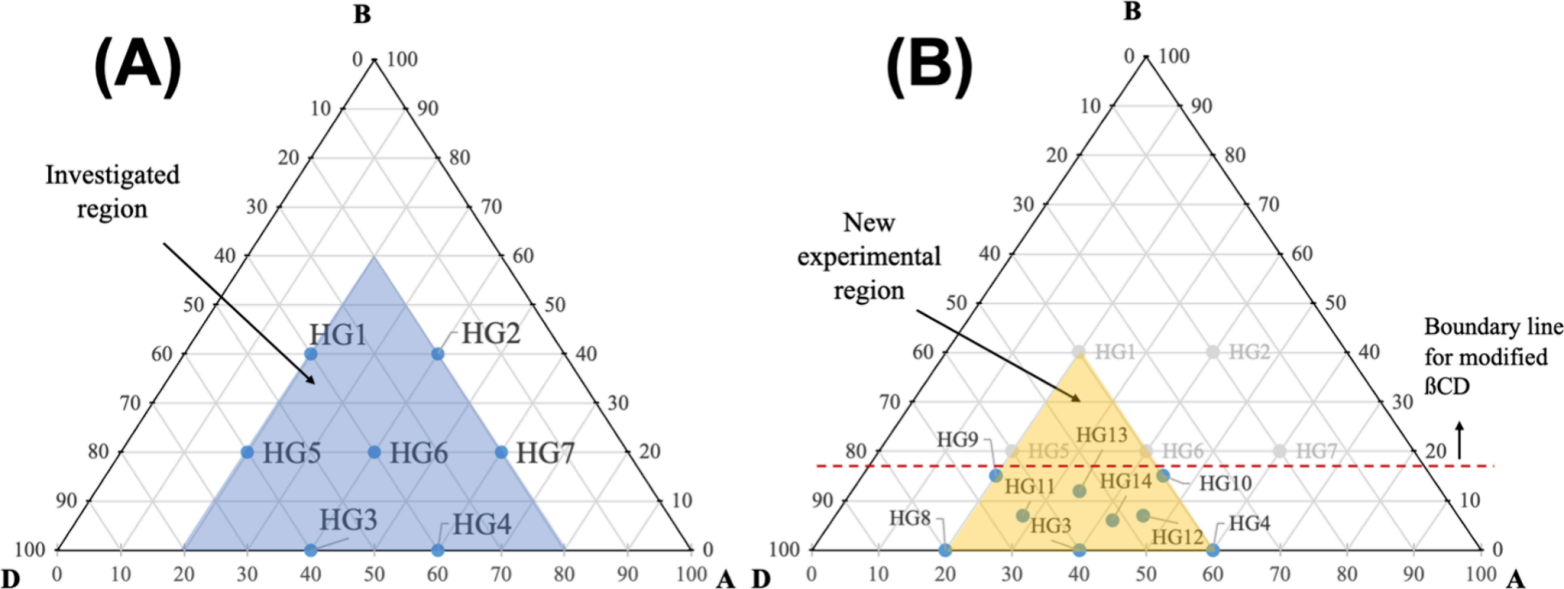
Composition design for the hydrogels: Simplex-centroid design (A), new experimental region after the first analysis (B).

The formulations HG1 to HG7 did not form hydrogels. It was observed, by a visual inspection, that the greater the βCD content, the lower the degree of cross-linking. Therefore, formulations from HG1 to HG7 had a viscous appearance. Based on these results, we constrained the maximum of βCD to 15% in weight, decreasing the experimental region. Then, new experiments were performed (HG9 to HG14) in an extreme vertex design (Figure 6B).^34^

Here, due to the constraints, the new investigated area was coded in terms of pseudocomponents as follows:

$$X_{coded}=\frac{X_{original}-a}{b-a}$$ 4

where *X*_*i*_, *a*, and *b* represent the composition (coded or original), the lower limit, and the upper limit from the hatched area, respectively. Thus, the sample composition (in coded variables) is given in Table 2.

**Table 2:** Proportions of Components (in Original and Coded Variables), and the Evaluated Response in the Simplex-Centroid Design

	original (%)			pseudocomponents			response^a^		
sample	D	B	A	D	B	A	S_pH1.2_	S_pH6.8_	ΔSwelling
HG3	60	0	40	0.50	0	0.50	20.57	32.87	12.30
HG4	40	0	60	0	0	1.00	22.00	64.75	42.75
HG8	80	0	20	1.00	0	0	11.11	20.89	9.78
HG9	65	15	20	0.625	0.375	0	10.91	14.88	3.97
HG10	40	15	45	0	0.375	0.625	5.97	9.06	3.09
HG11	65	7	28	0.625	0.175	0.200	13.62	21.09	7.47
HG12	47	7	46	0.175	0.175	0.650	12.06	26.02	13.96
HG13	54	12	34	0.350	0.300	0.350	4.38	7.81	3.43
HG14	52	6	42	0.300	0.150	0.550	5.84	11.52	5.68

a Results are the mean of three different experiments (*n* = 3).

The results from Table 2 were modeled by a quadratic model, which allowed the prediction of ΔSwelling values as a function of the composition in coded variables (eq 5). The analysis of variance (ANOVA) table and other statistics parameters is given in Supporting Information.

$$\Delta Swelling=-21.810X_{B}+42.130X_{A}+57.131X_{D}X_{B}-38.121X_{D}X_{A}-66.547X_{B}X_{A}$$ 5

Although the interaction of DMMAm with the βCD or acrylate cannot be neglected, the ΔSwelling is not directly dependent on the DMMAm content. Furthermore, due to the positive coefficient for A, we could conclude that the higher the acrylate content, the higher the ΔSwelling. The opposite effect is observed for βCD.

Interactions of the second order did not show a trend, indicating a complex relationship between the components in the hydrogel. Therefore, eq 5 was used to build a contoured surface to understand such a relationship. The result is shown in Figure 7.

**Figure 7:**
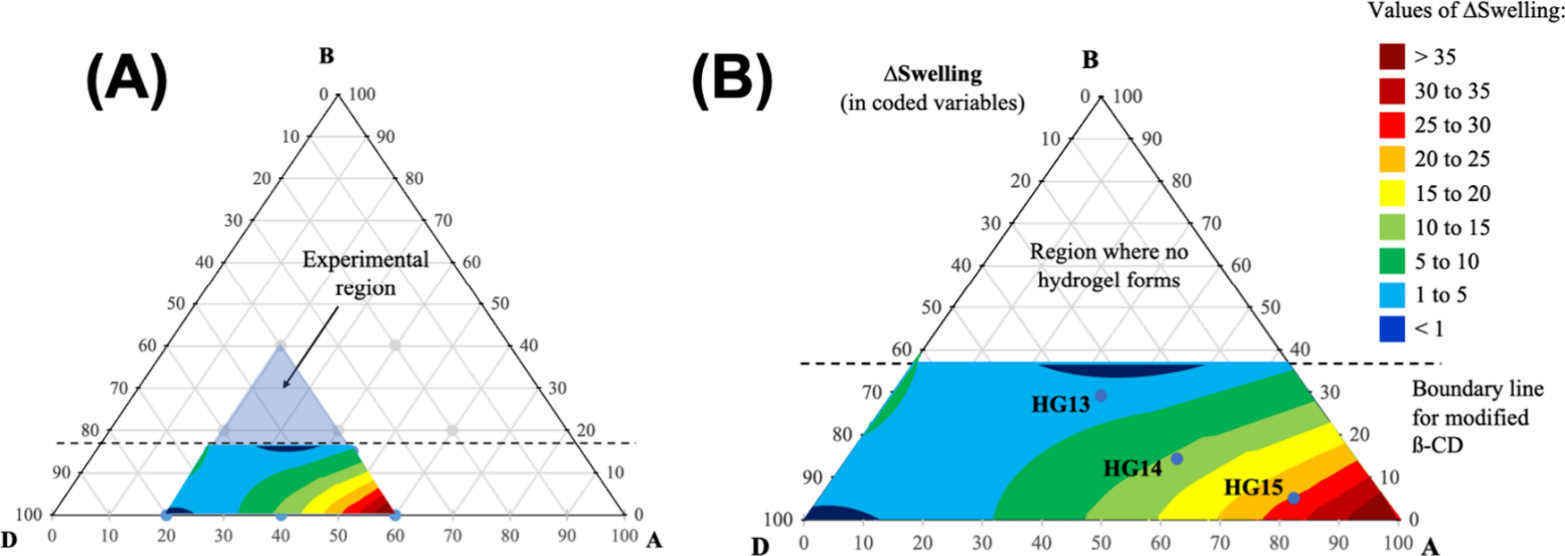
Contour surface obtained by the simplex-centroid design: Experimental region in original fraction weight (A), and experimental region in coded variables (B).

The surface response revealed two distinct regions where ΔSwelling is lower than 1.0 (in dark blue), indicating that the swelling is not pH-dependent. These regions fall in the highest concentration of βCD, and in the highest concentration of DMMAm.

Moving along the surface toward the acrylate content, the ΔSwelling increases. This effect can be understood by comparing samples HG4, HG3, and HG8. In such a case, the acrylate content is 60%, 40%, and 20%, respectively (Figure 8A). By increasing the acrylate content, the swelling increases in both pH. However, when the acrylate concentration exceeds 40%, a further increase enhances the absorption in pH 6.8. The COOH/COO^–^ equilibrium plays a key role in this behavior. For example, at pH 6.8, approximately 99% of COOH groups (p*K*_a_ ∼ 4.5) ionize to COO^–^, causing electrostatic repulsion, increasing the water affinity. On the other hand, under acidic conditions (pH 1.2), these groups are totally protonated, eliminating the electrostatic repulsion and reducing the swelling capacity.^12,35,36^

**Figure 8:**
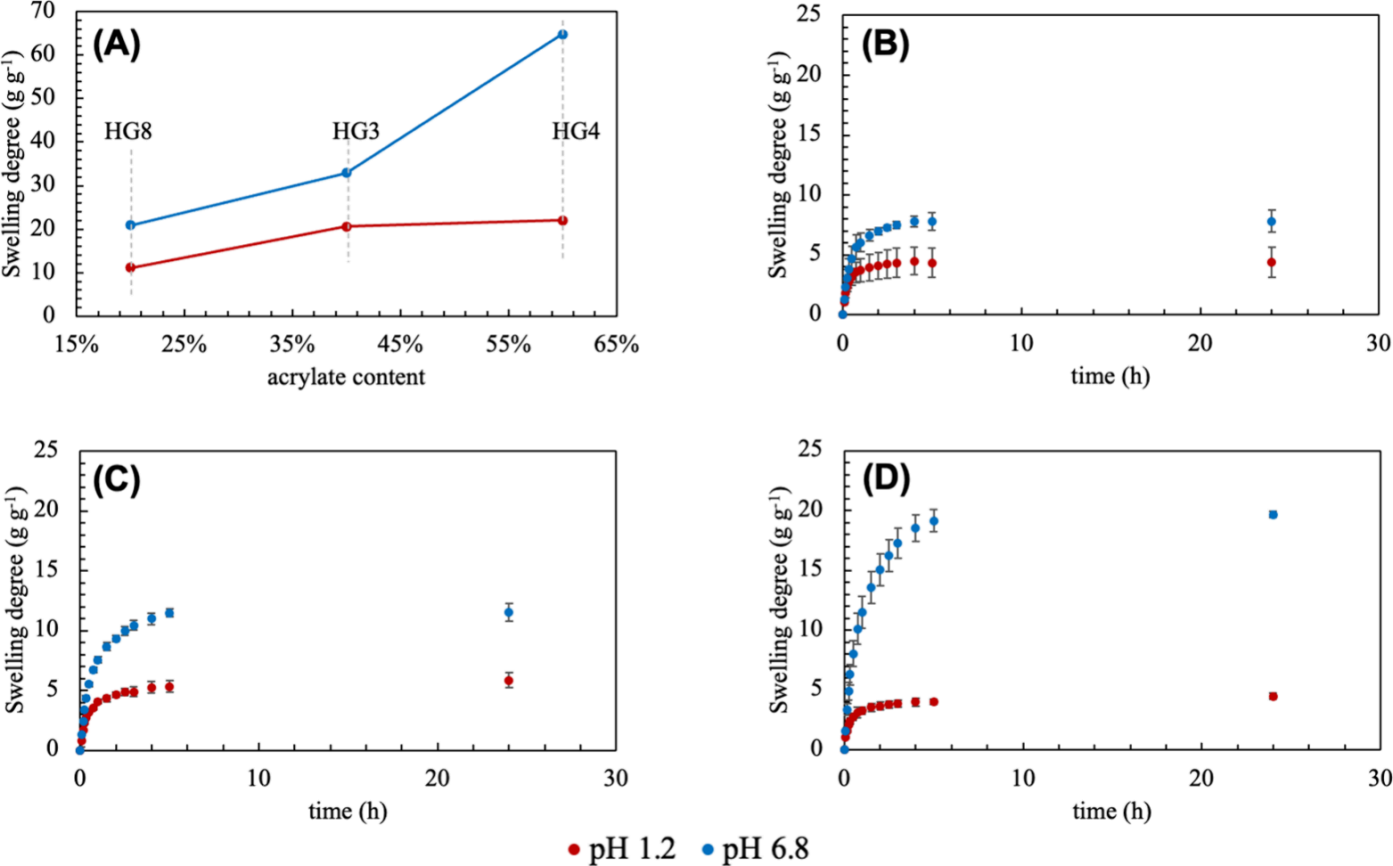
Effect of acrylate content on the swelling behavior in different pH (A), and swelling degree as a function of time for samples HG13 (B), HG14 (C), and HG15 (D).

For oral drug delivery excessive swelling in acidic conditions may cause premature release of HCO. Therefore, the ideal formulation must exhibit a high swelling degree in simulated intestinal fluid (SIF) and a lower swelling degree in simulated gastric fluid (SGF). The response surface methodology served as a predictive tool to identify formulations with the potential to achieve an optimal ΔSwelling, thereby guiding the selection of promising candidates for HCO release. Based on these criteria, and analyzing the data in Table 2, the samples HG13 and HG14 were selected for further study because showed the lowest swelling in SGF.

In addition, to validate our model, we synthesized an additional sample, HG15 (Table 3), with a relatively higher acrylate content (compared to HG13 and HG14) designed to achieve a ΔSwelling close to 25.

**Table 3:** Components Proportion Mixes (Original and Coded Variables) Synthesized for Validating the Quadratic Model^a^

	volume (μL)			original (%)			pseudocomponents		
sample	D	B	A	D	B	A	D	B	A
HG15	56	235	203	46	2	52	0.15	0.05	0.80

a Hydrogel synthesized with 500 μL of modified starch (0.10 g mL^–1^) and 6 μL of potassium persulfate (0.5 g mL^–1^).

The swelling behavior of samples HG13, HG14, and HG15 as a function of time is shown in Figures 8B to 8D. Regardless of the pH, samples reached swelling equilibrium within 4 h, indicating a consistent hydration process across different conditions. For all formulations, in simulated gastric fluid (SGF), the maximum swelling was approximately 5.0 suggesting that the acidic environment limited hydrogel expansion. As predicted by the mathematical model, sample HG15 exhibited the highest ΔSwelling among the tested formulations. However, the observed ΔSwelling was 15, lower than the predicted ΔSwelling of 25. This deviation suggests that additional factors, such as polymer interactions or network density, may have influenced the swelling behavior highlighting the need for further investigation. Nevertheless, the mathematical model was still able to predict a hydrogel formulation with the desired swelling profile (low swelling in SGF and enhanced swelling in SIF).

The swelling mechanism was analyzed using the Korsmeyer-Peppas model (eq 3), from which the diffusional exponent *n* was determined for samples HG13 to HG15 in SGF and SIF (Table 4). Assuming a thin-slab geometry and considering *n* < 0.5 as indicative of Fickian diffusion, a two-way ANOVA (factors: sample and pH, with 95% confidence level) on *n* showed a significant overall pH effect, with no notable effects of the sample or the sample-pH interaction. Posthoc one-way analyses within each sample revealed that pH did not significantly alter the swelling mechanism for HG13 and HG14. Their *n* values aligned with Fickian diffusion in both media. In contrast, HG15 showed a significant increase in *n* from SGF to SIF, indicating a shift from Fickian to anomalous transport at neutral pH. This anomalous transport suggests an increased contribution of macromolecular relaxation in the overall mechanism, consistent to the higher acrylate content.

**Table 4:** Diffusional Exponent *n* Obtained by the Power Law Equation and the Swelling Mechanism for Each Hydrogel

sample	*n* (SGF)	mechanism (SGF)	*n* (SIF)	mechanism (SIF)
HG13	0.44 ± 0.04^a^	Fickian diffusion	0.51 ± 0.04^a^	Fickian diffusion
HG14	0.38 ± 0.06^a^	Fickian diffusion	0.49 ± 0.02^a^	Fickian diffusion
HG15	0.44 ± 0.06^a^	Fickian diffusion	0.55 ± 0.01^b^	anomalous

The results are represented as the mean of three different experiments (mean ± SD). Superscript letters indicate statistically significant differences at a confidence level of 95%.

### 3.4 Scanning Electron Microscopy (SEM)

Figure 9 shows the SEM micrographs of the lyophilized hydrogels HG13, H14, and HG15, after achieving swelling equilibrium in SGF and SIF. All samples had a porous morphology characteristic of hydrogels. Moreover, hydrogels swollen in SIF exhibited a larger pore size. This porous structure facilitates controlled drug release by enabling water absorption and diffusion through the polymeric matrix.^4^

**Figure 9:**
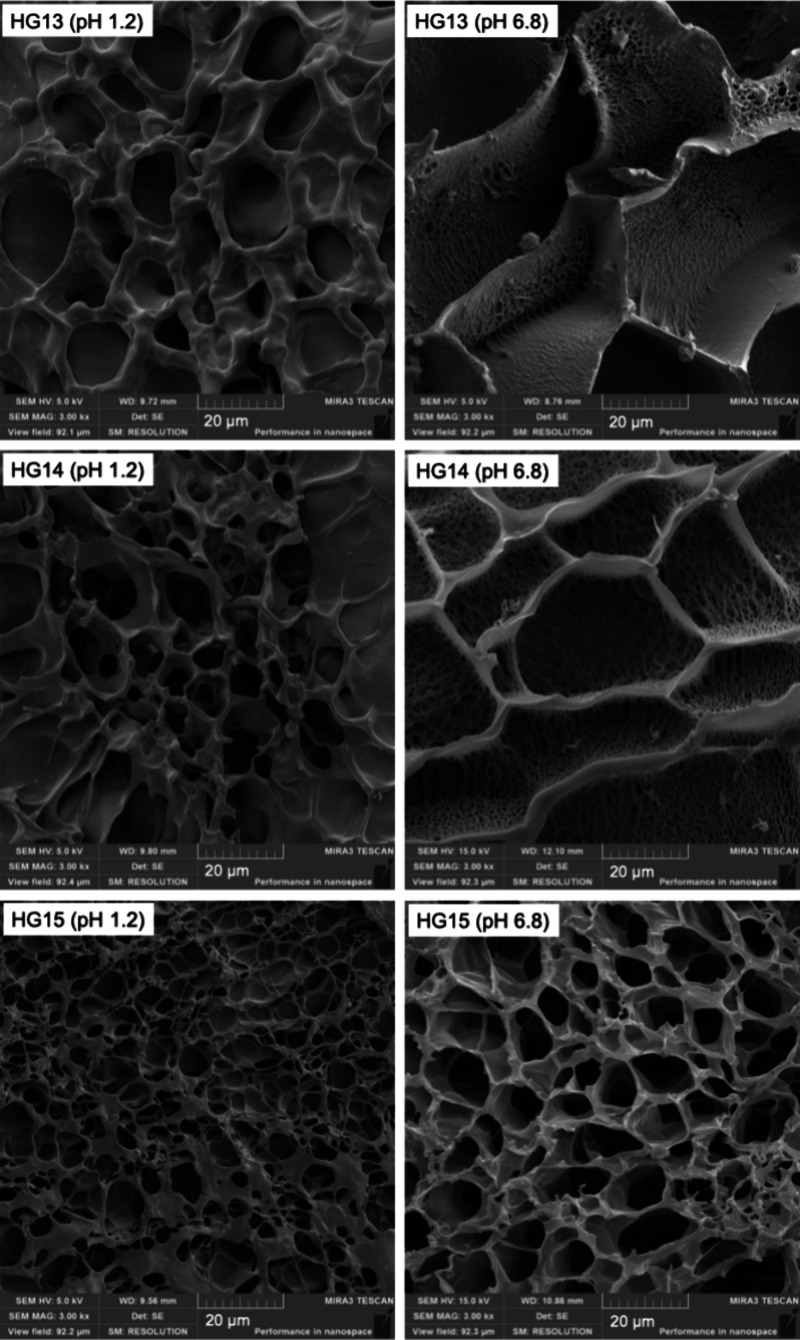
SEM images of HG13, HG14, and HG15 after swelling at pH 1.2 and 6.8.

Additionally, the hydrogels swollen in SIF exhibit a distinctive porous-in-porous architecture, where smaller pores are distributed within larger ones. This hierarchical porosity enhances the material’s ability to retain and transport fluids while providing an extended diffusion network for drug molecules. Furthermore, the stability of this porous network after the swelling process confirms the structural stability of the hydrogels, ensuring their integrity and functionality under physiological conditions.^37^

### 3.5 *In Vitro* HCO Release

The *In vitro* drug release curves for samples HG13, HG14, and HG15 are shown in Figure 10. Although we initially expected a correlation between pH-responsive swelling and HCO release, the release profiles did not follow the same trend. As highlighted in Section 3.3, increasing βCD content increases the contribution of macromolecular relaxation in the swelling mechanism; however, this was not directly reflected in the release kinetics. Among the tested samples, only HG14 exhibited a pH-responsive release behavior, suggesting that there may be an optimal concentration range of βCD for effective pH-responsiveness in drug delivery.

**Figure 10:**
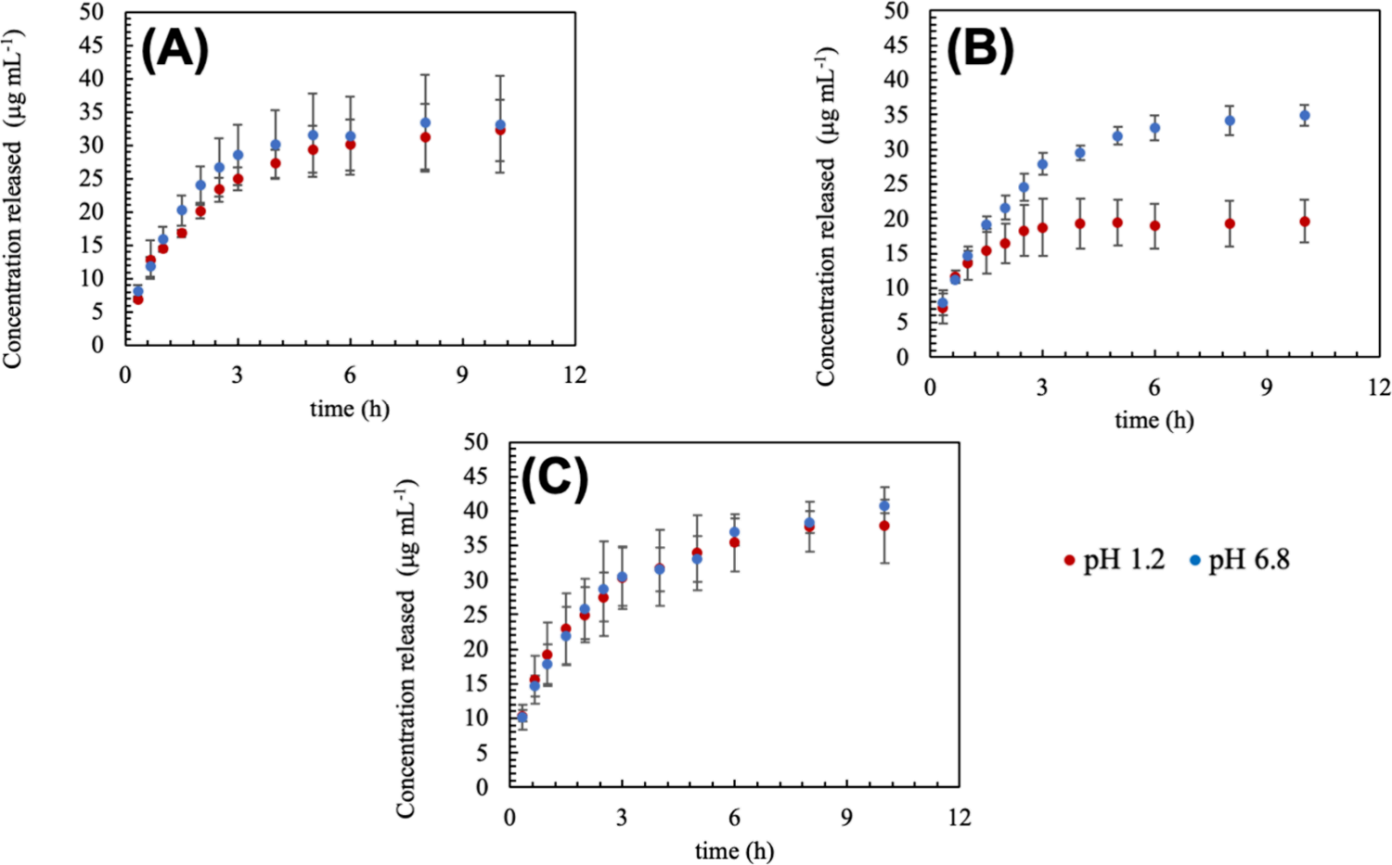
*In vitro* drug release of HCO as a function of pH from samples HG13 (A), HG14 (B), and HG15 (C).

To investigate the release mechanism, the experimental data were analyzed using the Korsmeyer-Peppas model (eq 3). This model defines the release mechanism based on the diffusional exponent (*n*). When *n* is 0.5, the mechanism release follows Fickian diffusion; when *n* is equal 1.0, it is governed by macromolecular relaxation; finally, an intermediate value is known anomalous transport, which have contribution of both the Fickian diffusion and the anomalous transport.^38^ The results are presented in Table 5.

**Table 5:** Diffusional Exponent from Fitting the Power-Law Equation (eq 3) to Drug Release Kinetics of Samples at Different pH

sample	pH 1.2	pH 6.8
HG13	0.60 ± 0.06^a^	0.60 ± 0.03^a^
HG14	0.46 ± 0.14^a,^ ^b^	0.58 ± 0.03^a,^ ^b^
HG15	0.47 ± 0.05^b^	0.52 ± 0.01^b^

The results are represented as the mean of three different experiments (mean ± SD). Superscript letters indicate statistically significant differences at a confidence level of 95%.

A two-way ANOVA (factors: sample and pH) performed on the *n* values showed no significant effect of pH, but a notable effect among samples. Thus, although sample HG14 exhibited pH-dependent release behavior, the release mechanism remained the same under both conditions (SGF and SIF). In addition, the Tukey test revealed that the release mechanisms differed significantly between samples HG13 and HG15. This result indicates that the *n* value for HCO release decreased with decreasing βCD content, which may be attributed to interactions between βCD and HCO. As the amount of βCD in the polymer matrix decreases, the contribution of polymer relaxation becomes insignificant, resulting in a release mechanism predominantly governed by Fickian diffusion.

Such a mechanism plays a crucial role in controlled drug release systems, allowing a gradual and sustained release of the active compound. This mechanism ensures that the drug is delivered at a controlled rate, reducing the risk of burst release and maintaining therapeutic concentrations over an extended period. Moreover, the porous structure promotes water absorption, swelling, and drug diffusion, making it an effective strategy for site-specific and time-controlled drug delivery.

## 4 Conclusions

Starch and β-cyclodextrin were successfully modified with GMA to introduce vinyl groups for further polymerization. The starch modification resulted in three different isomers: (i) one from the transesterification product and (ii) two from epoxide ring opening mechanisms. In contrast, βCD underwent a single modification via transesterification. This chemical modification influenced the affinity βCD for hydrocortisone, decreasing the binding strength, as confirmed in NMR analysis by a chemical shift in H3′ and H5′ protons.

The modified starch and modified βCD were copolymerized with sodium acrylate and DMMAm to synthesize hydrogels. The swelling behavior was optimized using a simplex-centroid experimental design. The contour surface allowed to identify an optimal hydrogel formulation with high swelling degree in SIF.

Sample HG14 was considered the most promising candidate for drug delivery application. Its drug release profile demonstrated pH sensitivity, releasing more HCO in simulated intestinal fluid. This result indicates that the hydrogel is suitable for intestinal drug delivery. The release mechanism trend to a Fickian diffusion, remaining consistent across different pH conditions. This mechanism ensures a controlled drug release rate, minimizing burst effects and maintaining therapeutic levels over time.

Additionally, the results revealed that modified βCD influences only the drug release mechanism and does not significantly affect the swelling behavior. Higher concentrations of βCD in the polymer matrix increased the contribution of macromolecular relaxation during HCO release, highlighting the role of host–guest interactions in modulating release kinetics.

These findings, the developed hydrogel system exhibits structural stability, effective swelling properties, and a well-regulated drug release profile, becoming an advanced material for controlled drug delivery applications.

## Supplementary Material



## Notes

2D ^1^H–^13^C HMBC spectrum of modified β-cyclodextrin; analysis of variance (ANOVA) table for the experimental response (ΔSwelling); residual plot comparing experimental and predicted values; time-dependent swelling curves at different pH values (37 °C) for each hydrogel sample; chemical shift variations indicating the inclusion of hydrocortisone (HCO) into the β-cyclodextrin cavity; determination of starch molecular weight by DOSY NMR (PDF)
