# Supplementary material for: Engineered Cyclodextrin-Starch Hydrogels for pH-Triggered Drug Release
Source: ACS Omega. 2025 Nov 5;10(45):54939–50. doi: 10.1021/acsomega.5c08972 (PMC12631703; doi:10.1021/acsomega.5c08972)
Supplement: Supplementary file 1 [file ao5c08972_si_001.pdf]

Engineered cyclodextrin-starch hydrogels for pH-triggered drug release

Adrielle C. Reis[a], Raphaela P. Guaringue[a], Vinicius M. Schaffka[a], Michele K. Lima-Tenório[a,b], Bárbara C. Fiorin[a], Adriano G. Viana[a], Ernandes T. Tenório-Neto[b,*]

a. Laboratory of Spectroscopy, Characterization and Modeling (LEsCaM), Department of Chemistry, State University of Ponta Grossa (UEPG), Av. General Carlos Cavalcanti, 4748, Ponta Grossa, Paraná, Brazil, CEP: 84030-900.

b. Laboratory of Spectroscopy, Characterization and Modeling (LEsCaM), Department of Chemistry, State University of Ponta Grossa (UEPG), Av. General Carlos Cavalcanti, 4748, Ponta Grossa, Paraná, Brazil, CEP: 84030-900.

* corresponding author: ernandes.tenorio@uepg.br

Support Information

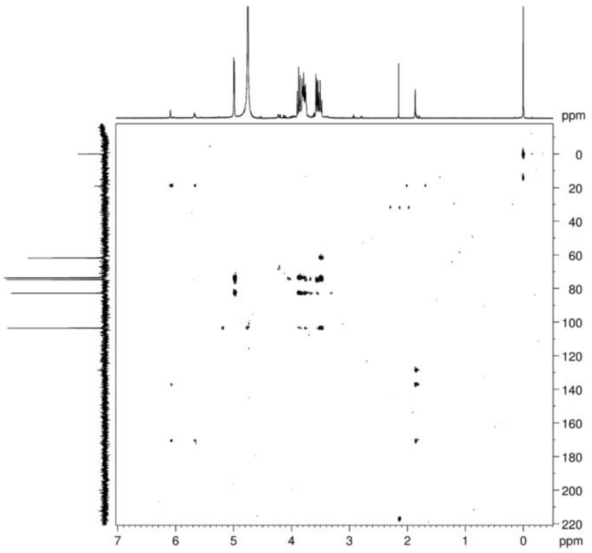

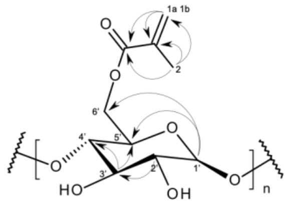

N	^{1}H	^{13}C	HMBC
1'	4.97 (*d*) *J*= 3.73 Hz	103.61	73.56; 61.95
2'	3,54 (*dd*) *J*= 10.13; 13.61 Hz	73.73	74.78
3'	3.86 (*dd*) *J*= 9.90; 18.98Hz	74.84	73.73; 82.87
4'	3.48 (*t*)	82.87	61.95; 73.56; 103.61
5'	3.75 (*m*)	73.56	-
6'	3,79 (*m*)	61.95	-
4	-	170.55	-
3	-	128.4	-
1	a - 6.07 (*q*)<br>b - 5.65 (*m*)	137.10	19.10; 137.10;170.55
2	1.84 (*dt*)	19.10	128.4; 137.10; 170.55

Figure S1. 2D ^{1}H-^{13}C HMBC spectrum of modified β-CD.

Table S1. Analysis of variance of the experimental response (ΔSwelling).

ANOVA	Sum of Squares	df	Mean Square	F-ratio	Tabulated F
Regression	1202.23	4	300.557	21.185	9.117
residue	45.562	3	14.187		
**Total**	1244.79	7			
**R^2**	0.9658				

df: degrees of freedom

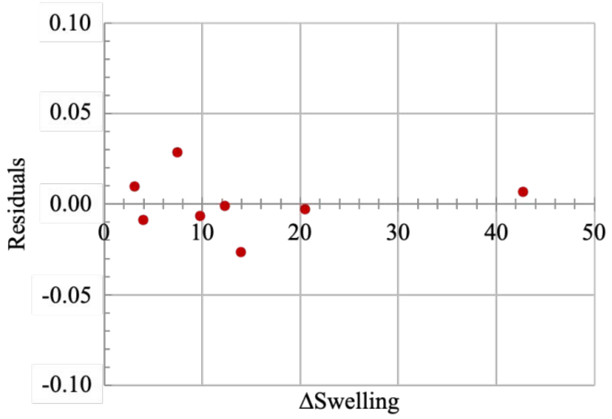

Figure S2. Residual plot comparing the experimental and the predicted results.

All parameters in Eq. (3) were significant, with a p-value < 0.05 confirming negligible residual, and the model is considered predictive. The determination coefficient (R^2 = 0.9999) indicates that 99.99% of the variation is accounted for by the model.

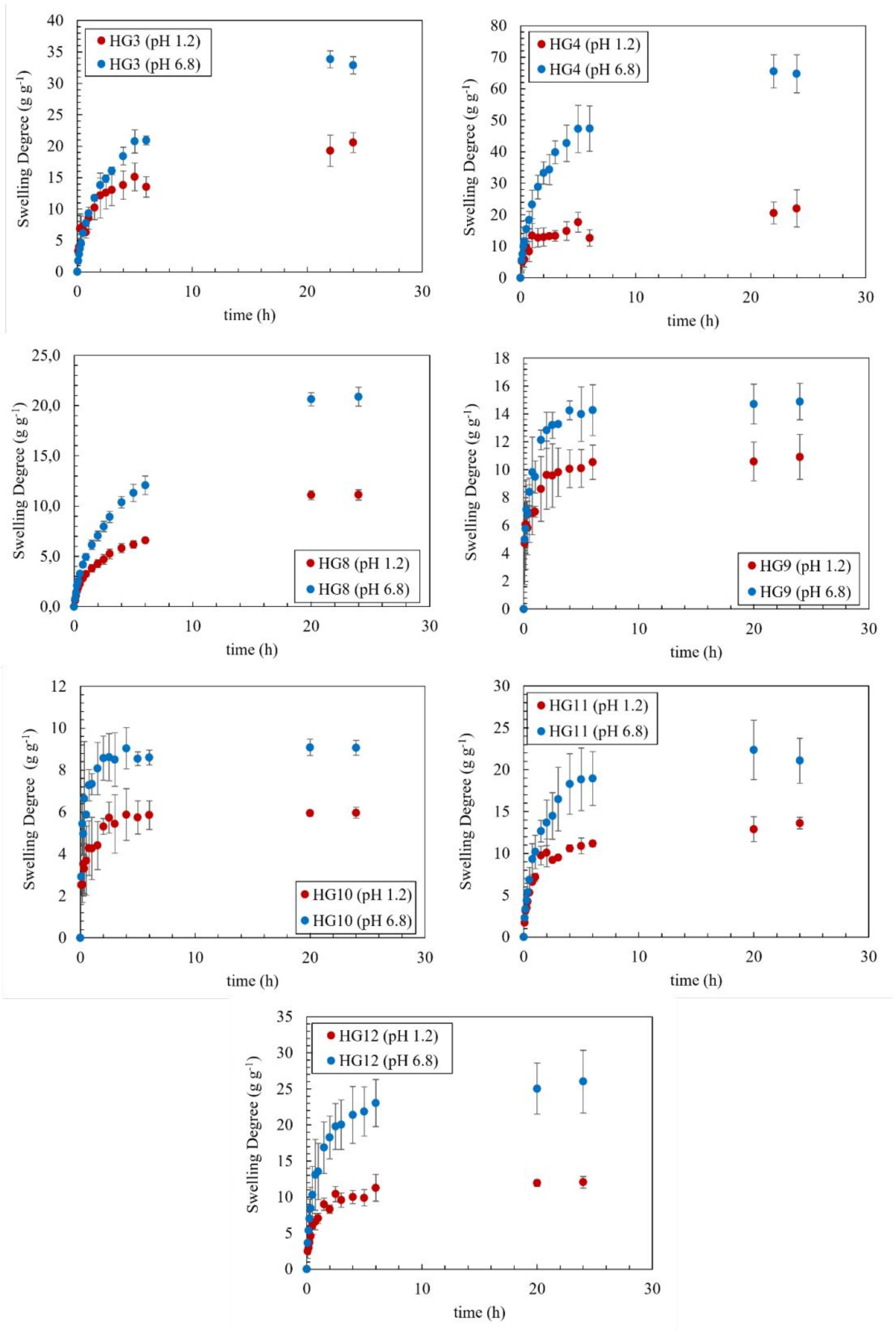

Figure S3. Time-dependent swelling curves at the indicated pH at 37 °C for each hydrogel sample.

All hydrogels analyzed demonstrated pH-responsive behavior,. The diffusion exponent values, ranging from 0.3 to 0.55, suggest that the water absorption mechanism is predominantly governed by Fickian diffusion, with no significant variations observed in response to changes in pH.

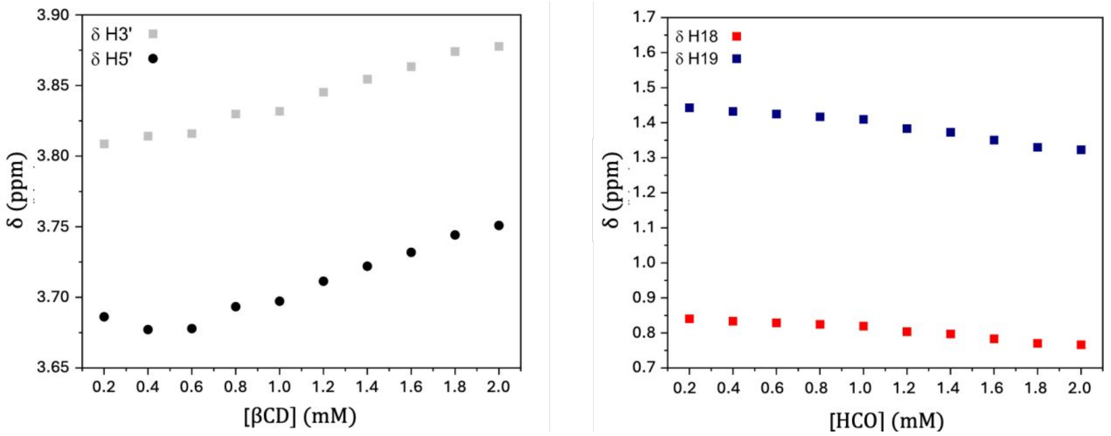

Figure S4. Chemical shifts variation indicating the inclusion of HCO into β-CD cavity.

Determination Molecular weight of Starch by DOSY

The average molecular weight of starch was estimated by Diffusion-ordered NMR spectroscopy (DOSY) using the method described by Viel S et al. Starch solution (1 mg mL^{-1}) was prepared in DMSO-d6, 0.05% TMS for the experiment. The DOSY NMR experiments were performed using the double-stimulated echo experiment and three spoil gradients (*dstegp3s*) to measure diffusion coefficients. The diffusion time (Δ) was 100 ms, with a gradient pulse duration (δ) of 2,5 ms. The gradient strength varied linearly from 25% to 95% in 32 steps. The number of scans was 16, with a relaxation delay of 1 s. The spectral width was the same as ^{1}H experiments. The experiments were conducted at 294.4 K. After Fourier transformation and baseline correction, the diffusion dimension of the DOSY spectra was processed by Bruker Dynamics Center software (version 2.8.8) (see supplementary material, Figure S5).

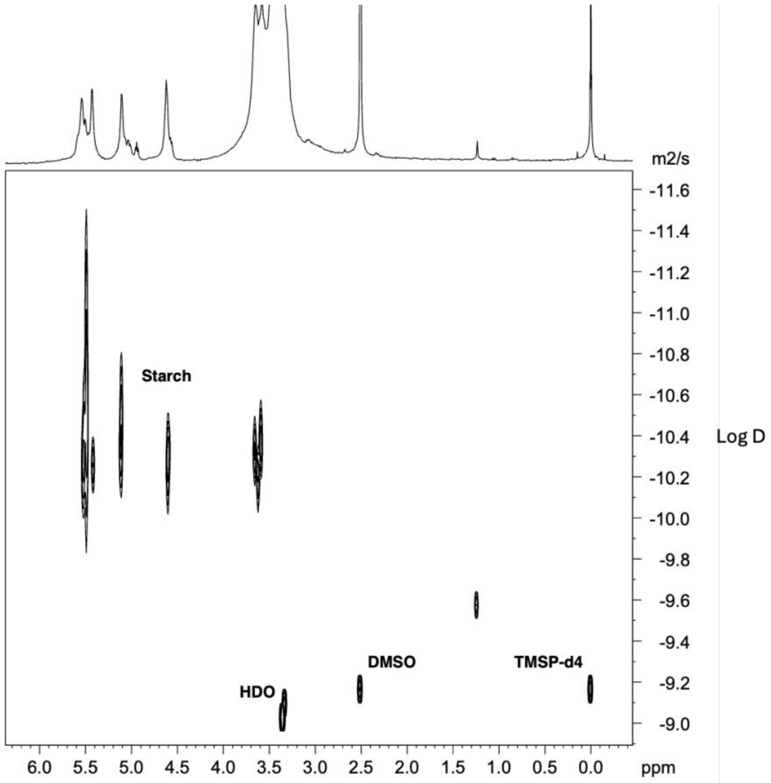

Figure S5. DOSY NMR spectra of a DMSO-d6 starch solution (1 mg mL^{-1}) at 294.4K.

The Stokes-Einstein equation, describes the diffusion coefficient:

$$D = \frac{k_B T}{6\pi\eta R_H}$$

where T, k_B, η, and R_H are the temperature, Boltzmann constant, the solvent viscosity, and the spherical particle of radius, respectively.

In the case of an ideal and monodisperse polymer:

$$R_H \approx aM^{\nu}$$

where a is a constant, M is the molecular weight, and ν is a parameter describing the relationship between the polymer and its solvent. In case of polymers the DOSY experiment will provide an average diffusion constant, and therefore an average molecular weight.

(Tooley, O., 2024) reported a solvent-independent universal calibration by using DOSY methodology for a non-charged polymers with Mw below 200 kDa:

$$M_w = 10^{\frac{(\log D + \log \eta) + 7.74 \pm 0.086}{-0.597 \pm 0.021}}$$

The bulk viscosity used was the reported by (Evans, R. et al., 2013) for deuterated solvents (2.19 mPa s). Therefore, the calculated molar weight was 10.57 ± 1.5 kDa

References:

Viel S. et al. Biomacromolecules (2003), 6: 1843-1847.

Tooley, O. et al. Macromolecular Rapid Communications (2024), 45: 2300692.

Evans, R. et al. Angewandte Chemie (2013), 52: 3199-3202.
